# G-protein Coupled Receptor Signaling in Pluripotent Stem Cell-derived Cardiovascular Cells: Implications for Disease Modeling

**DOI:** 10.3389/fcell.2015.00076

**Published:** 2015-12-09

**Authors:** Nazanin F. Dolatshad, Nicola Hellen, Richard J. Jabbour, Sian E. Harding, Gabor Földes

**Affiliations:** ^1^Myocardial Function, National Heart and Lung Institute, Imperial College LondonLondon, UK; ^2^The Heart and Vascular Center of Semmelweis University, Semmelweis UniversityBudapest, Hungary

**Keywords:** G-protein coupled receptor, Pluripotent stem cells, cardiovascular, disease modeling

## Abstract

Human pluripotent stem cell derivatives show promise as an *in vitro* platform to study a range of human cardiovascular diseases. A better understanding of the biology of stem cells and their cardiovascular derivatives will help to understand the strengths and limitations of this new model system. G-protein coupled receptors (GPCRs) are key regulators of stem cell maintenance and differentiation and have an important role in cardiovascular cell signaling. In this review, we will therefore describe the state of knowledge concerning the regulatory role of GPCRs in both the generation and function of pluripotent stem cell derived-cardiomyocytes, -endothelial, and -vascular smooth muscle cells. We will consider how far the *in vitro* disease models recapitulate authentic GPCR signaling and provide a useful basis for discovery of disease mechanisms or design of therapeutic strategies.

## General description of GPCRs

G-proteins are heterotrimeric proteins consisting of α, β, and γ subunits that can bind to both guanosine triphosphate (GTP) and guanosine diphosphate (GDP) nucleotides. G-protein-coupled receptors (GPCRs) are seven-transmembrane domain receptors (7TM receptors), which function through their interaction with G-proteins inside the cell. They can amplify extracellular signals to produce robust, varied, and cell-specific responses including chemotaxis, neurotransmission, cell growth, differentiation, and communication. GPCRs can bind a diverse range of ligands from large proteins to photons (Kristiansen, 2004) and also have a wide range of ligand-binding mechanisms (Gether et al., 2002). There are more than 800 GPCRs in the human genome, making it the largest receptor superfamily. GPCRs are divided into five distinct families using bioinformatic analysis: Glutamate, Rhodopsin, Adhesion, Frizzled, and Secretin (GRAFS classification system; Fredriksson et al., 2003; Gloriam et al., 2007). An up-to-date list of all human GPCRs as agreed by the International Union of Pharmacology subcommittee on Receptor Nomenclature and Drug Classification (NC-IUPHAR) can be found at http://www.guidetopharmacology.org/.

**Figure 1:**
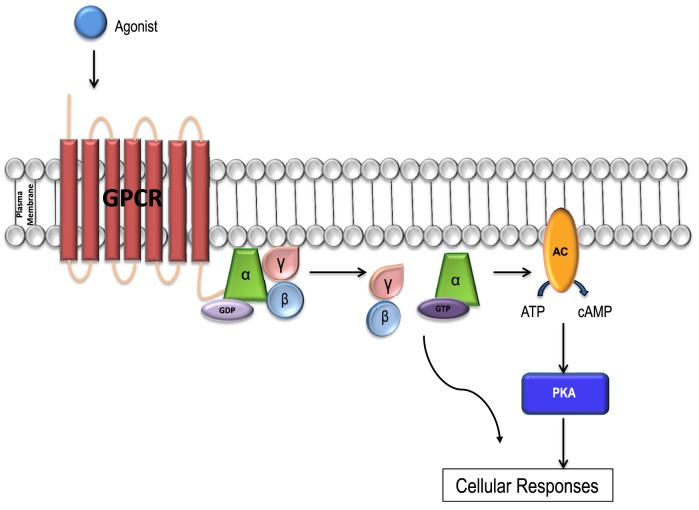
**GPCR signal transduction**. Each GPCR forms a complex with a unique Gα subunit. When the receptors are inactive the Gα subunit is inactive, bound to GDP and in a heterotrimeric conformation with βγ-subunits. The α and γ subunits are attached to the plasma membrane by lipid anchors. Once bound to a ligand, the receptor is activated and undergoes a conformational change, and the Gα subunit releases GDP, binds to GTP and is activated. The G_α_ subunit then releases the βγ complex leading to the activation of a variety of downstream effector molecules by the Gα subunit and βγ complex separately. The activated Gα subunit can bind to and activate a number of enzymes including adenylyl cyclase (AC) which catalyzes ATP into cAMP. Increases in the concentration of cAMP lead to the activation of the PKA enzyme which in turn activates the downstream signaling pathways resulting in a variety of cellular responses including glucose regulation and inotropy. The cycle is completed when G_α_-GTPase hydrolyzes GTP to GDP and becomes inactive. The G protein complex then re-couples the Gα with the G_βγ_ subunit.

GPCR signaling is activated via the receptor G-protein α-subunit, which can be divided into four major classes comprising of G_s_, G_i_, G_q_, and G_12∕13_, with each class consisting of multiple subtypes. To date 16 α subunits have been identified, with a total of 23 different isoforms. In addition, 5 β subunits and 12 γ subunits have also been identified in the human genome. There are multiple combinations of various isoforms that exist for each of the three G-protein subunits and the signaling pathways activated by them (Figure 1; Li et al., 2002; Tuteja, 2009).

While all GPCRs share common features like 7TM structure, interaction with the intracellular heterotrimeric G proteins and internalization, it is important to note that Frizzled (FZD) receptors which consist of 10 members and are classed separately in this superfamily do not all follow the same signaling mechanism as the other members and will be further discussed later (Foord et al., 2005; Gloriam et al., 2007).

Approximately 50–60% of all existing medicines are believed to target GPCRs (Fredriksson et al., 2003). By identifying the physiological role of GPCRs and their respective downstream signaling pathways, our understanding of many cardiovascular conditions has increased and new treatments have been developed. Indeed, two of the most prognostically important medications in treating heart failure target GPCRs and their pathways: (1) beta-adrenergic (β-AR) pathway blockers and (2) angiotensin converting enzyme (ACE) inhibitors/Angiotensin II receptor type 1 blockers (Kober et al., 1995; Salazar et al., 2007; Hunt et al., 2009; McMurray et al., 2012). Among an estimated 200 cardiac GPCRs (Salazar et al., 2007), drugs targeting adrenergic and angiotensin pathways alone account for the majority of prescriptions in cardiovascular diseases (Tang and Insel, 2004). In this review we aim to give an overview of the role of GPCRs in human pluripotent stem cells and their cardiovascular derivatives.

## GPCRs in human pluripotent stem cells (hPSC)

GPCRs exert a multitude of effects in pluripotent stem cells. A wide range of GPCRs are expressed in human embryonic stem cells (hESCs; Nakamura et al., 2009; Layden et al., 2010). Evidence exists for their roles in stem cell maintenance (Pébay et al., 2005; Inniss and Moore, 2006; Wong et al., 2007), pluripotency/self-renewal (Faherty et al., 2007; Kobayashi et al., 2010; Layden et al., 2010; Callihan et al., 2011), migration (McGrath et al., 1999; Miller et al., 2008) and survival (Jiang et al., 2007; Wong et al., 2007; summarized in Table 1). Less research however has been performed with human induced pluripotent stem cells (hiPSC).

**Table 1:** **GPCRs with roles in hESC/hiPSC and differentiation to cardiovascular derivatives**.

	**GPCR**	**Species**	**Cell type**	**References**
Maintenance and survival	S1P	Human	ESC	Pébay et al., 2005; Inniss and Moore, 2006; Wong et al., 2007
	LPA	Human	ESC	Dottori et al., 2008
	CB1 and CB2	Murine	ESC	Jiang et al., 2007
	CXCR4	Murine	ESC	Guo et al., 2005
Self renewal/pluripotency	FZD	Human	ESC	Sato et al., 2004; Cai et al., 2007; Melchior et al., 2008
Migration	CXCR4	Murine	ESC	Guo et al., 2005
Reprogramming to iPSC	FZD	Murine	iPSC	Marson et al., 2008; Li et al., 2011
		Human	iPSC	Li et al., 2009
Cardiac differentiation	FZD	Human	ESC/iPSC	Lian et al., 2012; Minami et al., 2012
	APJ	Human	ESC	Wang et al., 2012
	AT	Murine	ESC	Wu et al., 2013
Endothelial differentiation	FZD	Human	iPSC	Lippmann et al., 2012; Lian et al., 2014

### Maintenance and survival

GPCRs have an important role in stem cell maintenance. Lysophospholipid signaling, mediated by sphingosine-1-phosphate (S1P) and lysophosphatidic acid (LPA), control a wide range of cellular processes including stem cell maintenance via their respective GPCRs; S1P_1−5_ and LPA_1−5_. Signaling is mediated through phospholipase C (PLC), extracellular signal-regulated kinases 1/2 (ERK1/2), adenylate cyclase (AC), Ca^2+^ mobilization and activation of small GTPases. hESCs express both S1P_1−3_ and LPA_1−5_ (Pébay et al., 2005; Dottori et al., 2008). S1P in combination with platelet derived growth factor (PDGF) is responsible for the maintenance of hESC in an undifferentiated state via G_i_- and ERK-dependent mechanisms leading to the activation of pro-survival pathways, apoptosis inhibition and increased proliferation (Pébay et al., 2005; Inniss and Moore, 2006; Wong et al., 2007). From studies performed in murine ESC (mESC) expression of both CB1 and CB2 cannabinoid receptors have been detected and demonstrated to have a role in ESC survival (Jiang et al., 2007). Furthermore, the expression of the endocannabinoid receptor, 2-AG, may also contribute to ESC survival. The stromal cell derived factor 1 (SDF1 or CXCL12)/CXCR4 pathway which is widely known for its role in cell migration has also been found to enhance survival of mESC (Guo et al., 2005). To date, the role of these pathways has not been thoroughly investigated in hESCs/hiPSCs.

### Self-renewal/pluripotency

Expression and activation of G_s_- and G_i_-coupled GPCRs have been implicated in stem cell pluripotency in hESC and hiPSC (Nakamura et al., 2009). Colony morphology correlates closely with the maintenance of pluripotency. G_i_ inhibition with pertussis toxin (PTX) results in hiPSC/hESC colonies with a multi-layered appearance in contrast to a normal flat morphology, thereby preventing colony outgrowth (Nakamura et al., 2009). Proliferation, pluripotency and cell survival however, were unaffected by G_i_ inhibition. G_s_ activation on the other hand has been found to have no effect on colony morphology. While there is little evidence available in hPSCs, the activation of the G_αs_-cAMP signaling pathway in mESCs contributes to the maintenance of transcription factor expression which is important for pluripotency (Layden et al., 2010). In addition, evidence exists for the involvement of the cAMP/PKA pathway in mESC self-renewal pathways (Faherty et al., 2007).

**Figure 2:**
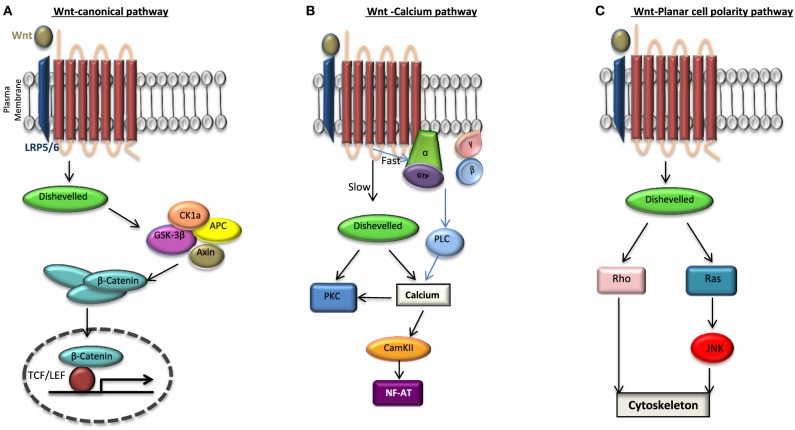
**Wnt signaling pathways**. When Frizzled receptor (FZD) is bound to its agonist Wnt it can activate one of the three pathways: **(A)** the canonical pathway in which the activation of disheveled (DVL) leads to the disassembly of the destruction complex; axin, adenomatosis polysis coli (APC), glycogen synthase kinase 3β (GSK3β) from β-catenin. This in turn increases the cytosolic level of β-catenin which is then translocated to the nucleus and binds to transcription factor T-cell factor/lymphoid enhancer factor (TCF/LEF) activating the transcription of target genes. β-catenin is phosphorylated by GSK3β and degraded when there is no Wnt activation of FZD. Non canonical pathways include **(B)** Wnt-Calcium pathway in which DVL activates protein kinase C (PKC) leading to the release of intracellular calcium thereby activating a calcium/calmodulin-dependent protein kinase II (CaMKII) and **(C)** Wnt-planar cell polarity pathway in which the activation of DVL signals to Rho GTPases (Rho or Rac or both). While Ras activation is signaled through the c-Jun amino (N)-terminal kinase (JNK), the activation of Rho-GTPases induces changes in the cytoskeleton. To date it has been found that Wnt signaling can not only lead to a direct activation of DVL independent of the heterotrimeric G proteins as seen in **(A)** but may also lead to a G protein-DVL dependent activation whereby DVL can bind to or become activated subsequently by the G proteins in the cell.

One of the key signaling pathways implicated in ESC self-renewal and pluripotency is the Wnt pathway. This signaling pathway can manifest in one of the three ways: (i) the canonical Wnt/β-catenin, (ii) Wnt/planar cell polarity (PCP) and (iii) Wnt/calcium pathways (Huang and Klein, 2004; Figure 2). Wnt ligands are lipid modified glycoproteins which bind to a Frizzled (FZD) receptors and a co-receptor; LRP 5/6. The co-receptor varies depending upon the signaling pathway.

In the past, while the Wnt/Ca^2+^ branch was recognized as the G protein-dependent pathway, the Wnt/ß-catenin and Wnt/PCP signaling pathways were regarded as heterotrimeric G protein-independent. However, many important studies have more recently shown that heterotrimeric G proteins play a more global role in the general Wnt signaling pathway (Katanaev et al., 2005; Egger-Adam and Katanaev, 2008). Since the two intracellular scaffolding proteins for FZD are heterotrimeric G proteins and Disheveled (DVL), what is still not clear in this field is the nature of this interaction and signaling following an agonist binding. Depending on the intermediary involvement of the DVL this interaction can be either direct or indirect. In a direct interaction FZD can act as either (a) a guanine nucleotide exchange factor (GEF), catalyzing the exchange of GDP for GTP on the G_αs_ subunit, releasing GTP-G_α_ and G_βγ_ or (b) a guanine dissociation inhibitor (GDI) sequestering GDP-G_α_ releasing G_βγ_. However, in the indirect model of interaction FZD may be acting through a higher order complex consisting of FZD, DVL, and the heterotrimeric G protein complex, whereby the FZD-G_α_G_β_G_γ_ binding and signaling is via DVL (Klipp and Liebermeister, 2006; Schulte and Bryja, 2007; Nichols et al., 2013).

Expression of the pluripotency genes Sox-2, Nanog, OCT3/4 and brachyury are targets of the Wnt/β-catenin pathway (Sokol, 2011). hESCs have been shown to express Wnt1, members of the frizzled receptor family FZD_1, 3−6_ and secreted frizzled related protein (SFRP) family (SFRP1, SFRP2, FRZB, SFRP4; Walsh and Andrews, 2003). The SFRP family act as antagonists to the Wnt pathway. There is contrasting evidence to support the role of Wnt signaling in hESC. Wnt3a and Wnt1 have been shown to stimulate hESC proliferation and maintain pluripotency via the canonical Wnt signaling pathway (Sato et al., 2004; Cai et al., 2007). In contrast, Wnt/β-catenin activation did not maintain the undifferentiated and pluripotent state of hESC (Dravid et al., 2005). The Wnt receptor FZD_7_ has also been identified as important for hESC maintenance and self-renewal. Loss of OCT-4 expression and alterations to colony morphology were observed after knockdown of FZD_7_ (Melchior et al., 2008).

### Cell migration

In addition to contributing to stem cell maintenance, the SDF1/CXCR4 pathway is important in endogenous stem cell trafficking during embryogenesis (McGrath et al., 1999; Miller et al., 2008). Activation of CXCR4 by SDF1 stimulates a number of pathways involved in motility, chemotaxis, adhesion and secretion, via activation of a number of signaling cascades including: adhesion, PI-3K-AKT, MEK-MAPK p42/44, and JAK/STAT (reviewed in Kucia et al., 2004). Both SDF1 and CXCR4 expression have been detected in mESCs and have been shown to be chemotactic for these cells (Guo et al., 2005). Priming of this pathway with sphingosine 1 phosphate (S1P) prior to the transplantation of cells enhanced cardiac and vascular remodeling in a rat model of pulmonary arterial hypertension (Kang et al., 2015). At the time of writing this review no evidence existed for the expression of SDF/CXCR4 in hESC or hiPSC.

### Reprogramming somatic cells to iPSC

The modulation of the Wnt signaling pathway appears to play a role in reprogramming. Inhibition of the downstream signaling molecule, GSK3, has the potential to replace Klf4 in the Yamanaka reprogramming cocktail in murine cells (Lyssiotis et al., 2009). The use of a combination of different small molecules, including the GSK3β inhibitor CHIR99021, has been reported to induced murine iPSC reprogramming in the presence of a single transcription factor (OCT4; Li et al., 2011). Additionally, CHIR99021 in combination with an inhibitor of lysine-specific demethylase, has been used in the presence of OCT4 and Klf4 to successfully reprogram human keratinocytes (Li et al., 2009). Application of Wnt3a has been reported to enhance reprogramming in conjunction with Klf4/OCT4/SOX2 in the absence of c-myc (Marson et al., 2008). A cocktail of small molecules, including the cAMP activator forskolin, has also been shown to have the potential to replace OCT4 during murine iPSC-reprogramming (Hou et al., 2013). In addition to the expression of the Yamanaka transcription factors, morphological changes are necessary for cellular reprogramming.

## GPCRs in cardiovascular differentiation

GPCRs have a fundamental role in early and late mesoderm formation during development and cardiovascular cell differentiation. To date, there are three platforms for cardiovascular cell differentiation: monolayer, embryoid body (EB) and microcarrier cultures. The efficiency of these methods depends on several factors including: (a) the biomolecules used (growth factors or small molecule inhibitors), (b) the condition of the hPSC culture expansion, and (c) the activation or deactivation time of molecular signals in guiding the differentiation toward cardiovascular lineages (Chen et al., 2014). Differentiation is achieved through the coordination of diverse molecular pathways. Elucidation of the complex molecular signals that are evoked during hPSC differentiation have enabled specific targeting of their activities to enhance cell differentiation and promote tissue regeneration.

Earlier protocols for the production of cardiomyocytes (CMs; Xu et al., 2002; Zhang et al., 2009), endothelial cells (ECs; Goldman et al., 2009; Földes et al., 2010) and vascular smooth muscle cells (VSMCs; Ge et al., 2012; Dash et al., 2015) relied on EB formation, whereby hPSC undergo spontaneous differentiation following the formation of 3D, non-adherent structures. Although this method generated cells of the required lineages, it was relatively inefficient. Differentiation has been much improved in recent years with use of factors found to be involved in mesoderm formation *in vivo*. GPCR Wnt signaling molecules and non-GPCR fibroblast growth factor (FGF), bone morphogenetic protein 4 (BMP4) and Activin A are all widely used for cardiomyocyte differentiation (Laflamme et al., 2007; Yang et al., 2008). In all protocols, the concentrations and duration of each treatment depends on the platform as well as the hPSC line in use.

For endothelial cell differentiation growth factors frequently utilize FGF2, which has been shown to promote the formation of endothelial progenitors (Evseenko et al., 2010), and BMP-4, which acts to accelerate commitment to the endothelial lineage (Goldman et al., 2009). Amongst the key pro-angiogenic growth factors, vascular endothelial growth factor (VEGF) is arguably the most important and has been demonstrated by multiple studies to dramatically increase the yield of ECs during differentiation (Tatsumi et al., 2011; Adams et al., 2013). GPCR agonists such as thrombin and angiotensin II can directly modulate vascular remodeling and they can also act indirectly through the induction of VEGF (Richard et al., 2001).

Various differentiation protocols have been validated and replicated to differentiate pluripotent stem cells into vascular smooth muscle (VSMC) like cells with mature characteristics displaying cellular markers (smooth muscle α-actin, calponin) and an adult morphology (fibrous). In addition, they display similar contractile responses to agonists such as carbachol (Ge et al., 2012; Karamariti et al., 2013; Wanjare et al., 2013; Sinha et al., 2014; Dash et al., 2015). For generating smooth muscle cells, various limitations using the EB method of differentiation led researchers to develop improved protocols of VSMC differentiation from hiPSC using monolayers of extracellular matrix (ECM) proteins in the presence of PDGF subunit B homodimer (PDGF-BB) and transforming growth factor beta (TGF-β; Karamariti et al., 2013; Wanjare et al., 2013) and heparin (Bajpai et al., 2012).

### Frizzled receptor

Wnt signaling is necessary for different steps of the cardiac development in embryonic stem cells, including myocardial specification, cardiac morphogenesis, and cardiac valve formation (Korkaya et al., 2009). It is believed the non-canonical Wnt pathway plays a key role in cardiac morphogenesis and affects the specification and expansion of cardiac progenitor cells (Korkaya et al., 2009). Hence, most of the latest protocols in the differentiation of hPSCs to CMs involve the use of various Wnt inhibitors and downstream molecules like GSK3β.

Of the three stages of cardiomyocyte differentiation: mesodermal induction, cardiac progenitor generation and cardiomyocyte generation and maintenance, the initial step of mesoderm induction is induced by the activation of the TGF-β pathway (Watabe and Miyazono, 2009; Xu, 2012). This can be achieved by the use of growth factors, BMP4 and Activin A. An indirect activation of the TGF-β signaling pathway has been performed *in vitro* by using small molecules, such as GSK3β inhibitors (CHIR99021 or BIO) which have an increasing effect on the endogenous levels of BMP2/4 (Lian et al., 2012; Minami et al., 2012). For the second stage of cardiac progenitor induction the TGF-β pathway has to be inactivated. This can be achieved by: (a) the removal of the activators and addition of growth factors including FGF2 and/or VEGF, which activate the ERK signaling pathway, or (b) the addition of small molecule Wnt inhibitors (KY02111, XAV939, DKK1, IWP-2, and IWR-1; Chen et al., 2006). This results in the formation of the cardiac progenitor lineage from mesodermal cells and inhibits the development of smooth muscle and endothelial cell lineages (Woll et al., 2008; Yang et al., 2008). The final stage of CM generation and maintenance, which takes place from day 8 is also found to be dependent on the inhibition of the Wnt/β-catenin signaling pathway (Gessert and Kühl, 2010). It can therefore be concluded that Wnt signaling plays a biphasic role in human cardiogenesis, being both activated during the early phase and inhibited during the late phase of cardiac differentiation (Lian et al., 2012).

During fetal growth the compact myocardium proliferates more rapidly when compared to the trabecular myocardium in luminal regions of the heart (Jeter and Cameron, 1971; Luxán et al., 2013). The proliferation of fetal cardiomyocytes in this region is necessary for the correct morphogenesis of ventricular myocardium, trabeculae, and chamber cavities. It has recently been shown that this regional expansion of ventricular myocytes is regulated by the Wnt/β-catenin pathway. The increase in the ventricular proliferation is maintained until birth. This fetal Wnt signaling pathway is re-expressed upon myocardial infarction and induced ischemic heart injury in mice (Buikema et al., 2013a,b). Hence, it has been suggested that in adult myocardium Wnt/β-catenin may play a role in endogenous cardiac repair; however, the exact role of this pathway in the adult cardiac homeostasis is not yet known (Oka et al., 2007; Oerlemans et al., 2010).

In addition, the production of pluripotent stem cell-derived endothelial cells (PSC-EC) has also been shown to be dependent on small molecule activation of canonical Wnt signaling. This was demonstrated to be an effective mechanism using a 2D culture system, even in the absence of exogenous VEGF (Lian et al., 2014). The canonical Wnt ligands, Wnt7a and Wnt7b, have been implicated in blood-brain barrier (BBB) development *in vivo* (Daneman et al., 2009). In order to generate human BBB-ECs, the Wnt pathway was targeted in differentiating hPSCs (Lippmann et al., 2012). A Wnt target gene called Stimulated by retinoic acid 6 (STRA6) which acts as a vitamin A transporter is found in the BBB (Szeto et al., 2001). It is highly expressed in adult brain ECs in comparison to lung or liver cells, and is up-regulated during the course of BBB cell differentiation (Lippmann et al., 2012).

### Angiotensin receptor

Angiotensin receptors are members of the GPCR family and are composed of two main types; angiotensin receptors I and II (AT_1_ and AT_2_) which exhibit similar affinities for angiotensin II (Ang II; de Gasparo et al., 2000). The activated AT_1_ binds to G_q∕11_ and G_i∕o_ to activate phospholipase C and increase the cytosolic Ca^2+^ concentration, whilst AT_2_ exerts its effect via coupling to the G_i2∕3_ components of the heterotrimeric G-proteins (Higuchi et al., 2007). Activated AT_1_ and AT_2_ have mutually counteracting hemodynamic effects in the cardiovascular system. AT_1_ is believed to be responsible for the contractile response while AT_2_ is involved in the relaxation response to Ang II (Batenburg et al., 2004). Ang II promotes the differentiation of mESC-CM through AT_1_ (Wu et al., 2013). Currently no role in human cardiovascular differentiation has been described. AT_1_ and AT_2_ are expressed on human hemangioblasts. The differentiation into endothelial progenitors can be influenced by modulating the signaling through these receptors. ACE activity is required for hemangioblast expansion. AT_1_- or AT_2_ specific inhibitors dramatically augment endothelial differentiation (Zambidis et al., 2008).

### Apelin receptor

This receptor, also known as Angiotensin receptor like 1 (AGTRL1 or APJ) is a member of the GPCR family that binds apelin (APLN; Tatemoto et al., 1998; Lee et al., 2000) and ELABELA/Toddler (Chng et al., 2013; Pauli et al., 2014). APJ is coupled to G_i_ and/or G_q_ and is expressed in the mesodermal cells of the secondary heart field in mouse embryo. It couples extracellular signaling with chromatin modifications in pluripotent stem cell cardiomyogenesis (D'Aniello et al., 2013). During hESC differentiation, APJ marks mesodermal precursors (Vodyanik et al., 2010). While on adult cardiomyocytes, the expression of this receptor is a potent regulator of contractility (Szokodi et al., 2002; Berry et al., 2004; Ashley et al., 2005); on early embryonic cells it is believed to regulate the migration of progenitor cells fated for cardiomyocyte differentiation (Scott et al., 2007; Zeng et al., 2007). Hence, Apelin has been used in the differentiation of both mouse and human ESCs to cardiomyocytes in combination with mesodermal differentiation factors including BMP4, bFGF, and Activin A. Using an EB differentiation method and by administering these factors in a specific temporal sequence, it has been shown that apelin can indeed promote cardiac differentiation and lead to earlier beating EBs when compared to controls (Wang et al., 2015). We and others have shown that APJ and one of its ligands apelin have an important regulatory role in angiogenesis (Scott et al., 2007). A second ligand elabela (or *Toddler*) has been recently discovered which is required for the normal development of vasculature through activation of APJ. Elabela/APJ signaling pathway was shown to be functional in the human system as well (Chng et al., 2013; Wang et al., 2015). To date, no published data is available for the new ligand in hPSCs.

### Lysophospholipid signaling

Lysophospholipid signaling is important for vascular development and maturation, but *in vitro* stem cell models are currently lacking. Knockout mice of S1P_1_ (G_i_-coupled receptor for sphingosine-1 phosphate) has been showing high lethality at E12.5 (Soriano, 1999). This has been attributed to the necessary function of ECs (Kataoka et al., 2003) and the receptor has also been found to be essential for vascular maturation (Liu et al., 2000). Furthermore, *in vivo* studies have shown that S1P protein synergizes with FGF-2 and VEGF in angiogenesis and vascular maturation through S1P_1_ (Garcia et al., 2001). While S1P_1_ couples directly to the G_i_ pathway, the other receptor isoforms known also as endothelial differentiation gene 3, 5 (Edg-3 and -5) stimulate G_i_, G_q_, and G_13_ pathways (Ancellin and Hla, 1999; Windh et al., 1999).

### Protease-activated receptor-1

Protease-activated receptor-1 (PAR-1) is one of the four members of the PAR subfamily of GPCRs, which are highly expressed in platelets as well as ECs, myocytes, and neurons (Macfarlane et al., 2001). PAR-1 is activated by serine proteases including thrombin, whereby the N-terminus of the receptor is cleaved and this in turn acts a tethered ligand activating the receptor. As PARs are involved in maintaining hemostasis and thrombus formation in atherosclerotic vessels, these are being tested as drug targets (Sambrano et al., 2001). As a member of the receptor family, PAR-1 was shown to play a role in embryonic development (Griffin et al., 2001), partially via modulation of downstream signaling proteins such as the heteromeric G-protein subunit G_α13_ (Ruppel et al., 2005). The role in hPSC differentiation remains to be defined.

### Adrenergic receptors

Adrenergic receptors can be broadly divided into alpha (α-AR) and beta (β-AR) receptors. The β-ARs have been shown to have a role in cardiomyocyte differentiation (Yan et al., 2011). β_1_-ARs couple to stimulatory G proteins (G_s_). Once stimulated, G_s_-proteins interact with the enzyme adenylyl cyclase (AC), which in turn increases the production of cAMP. β_2_-ARs and β_3_-ARs can also couple to the inhibitory G (G_i_) protein (Gauthier et al., 1996; Gong et al., 2000). β_2_-AR G_i_ pathways decrease AC activation and cAMP production as well as the downstream phosphorylation of cardiac proteins including troponin I, myosin-binding protein C and L-type calcium channels. The net result opposes the action of the G_s_ resulting in reduced contraction of the cardiac myocytes (Xiao et al., 1995; Woo and Xiao, 2012). Further studies in mESCs have shown that β-ARs play a role in ESC-CM differentiation via ERK and p38 activation using β-AR agonists. β_1_-ARs and β_2_-ARs have been found at different stages of cardiac differentiation both at mRNA and protein levels. The expression of β_1_-AR is lower than β_2_-AR until day 7. After day 7 it increases gradually, reaching a peak at day 14, and remains at a high level until day 21. In contrast, β_2_-AR is expressed at a high level even before differentiation, with no obvious change after inducing cardiac differentiation. It is therefore believed that β_2_-AR might be the predominant subtype during the early stage of differentiation, while β_1_-AR might be the predominant subtype for the late stage of cardiac differentiation (Yan et al., 2011).

## GPCRs in pluripotent stem cell cardiovascular derivatives

In recent years, the ability to derive human cardiovascular cells from pluripotent stem cells, which have unlimited renewal capacity, has generated considerable interest. hPSC-derived cardiovascular derivatives have the potential to reduce the use of animal models and provide more physiologically relevant models of disease. They can be produced in quantities that are suitable for use in medium to high throughput screens, and platforms are being developed to measure their various functional outputs, including calcium transients, contraction, tubule formation, and cytotoxicity/signaling (Mioulane et al., 2012; Mercola et al., 2013; Stoehr et al., 2014; Simons et al., 2015). The relative ease of production and commercial availability further enhances their appeal for pharmaceutical screening and organ repair.

### Human pluripotent stem cell-derived cardiomyocytes

#### Alpha-adrenergic receptors

α-ARs (α_1a_, α_1b_, α_1d_) regulate the cardiovascular system by activating the G_αq_ pathway. Once activated, G_αq_ activates phospholipase C (PLC), which causes an increased myo-inositol-1,4,5-trisphosphate level and subsequent increase in calcium release from the endoplasmic reticulum (Exton, 1985; Salazar et al., 2007). These receptors are primarily thought to regulate blood pressure, inotropy and hypertrophy by cross talk between the α-AR subtypes and also with β-ARs (Salazar et al., 2007). For example, overexpression of the α_1a_-AR increases cardiac contraction but not hypertrophy (Lin et al., 2001) and overexpression of α_1b_-AR results in a decreased response to β-AR stimulation by isoprenaline left ventricular (LV) contractility, potentially as a result of additional G_i_ coupling (Akhter et al., 1997). Additionally, a deficiency in the α_1b_-AR receptor results in a blunted blood pressure response to the α_1_-AR receptor agonist phenylephrine (PE; Cavalli et al., 1997). Alpha-2-adrenergic receptors (α_2_-AR) are G_i_-coupled receptors and oppose the action of G_s_ signaling by inhibiting AC and therefore cAMP production and the various downstream sequelae (Salazar et al., 2007). α_2_-ARs are presynaptic and suppress presynaptic noradrenaline release and their role is to oppose the sympathetic stimulation of β_1_-AR, β_2_-AR, and α_1_-ARs during increased adrenergic stimulation. Their importance was shown with higher incidences of heart failure in patients with genetic polymorphisms resulting in the loss of function of α_2_-ARs (Small et al., 2002).

The expression of α-ARs in hPSC-CMs, as reported by our own group, shows an early transient up-regulation during differentiation followed by a rapid stable down-regulation of ADRA1A in hiPSC-CM and hESC-CM. Conversely ADRA1B was found to be increased in an apparently compensatory manner. Other subtypes of α-AR namely ADRA1D and ADRA2C have also been shown to be present in these cells, but the overall expression of these receptors and their G-proteins; G_q_, G_β1_, and G_γ2_ is believed to be insufficient for the hypertrophic response to PE (Földes et al., 2014). However, when investigating the exact localization of α_1_-ARs in adult cardiomyocytes, recent studies found them expressed in the nuclei rather than just the sarcolemma itself: this may contribute to the differences observed in the response levels of various cell types to PE (Wu and O'Connell, 2015).

#### Beta-adrenergic receptors

There are three main types of β-ARs present in the human cardiovascular system; β_1_-ARs are the most abundant accounting for 75–80% in healthy human hearts (Rockman et al., 2002); with β_2_-ARs and β_3_-ARs making up the remainder. β_1_-ARs primarily modulate the inotropic and chronotropic responses of the human heart. Once activated, stimulatory G_s_-proteins interact with AC, which in turn increases the production of cAMP. The increased levels of cAMP result in increased binding to protein kinase A (PKA) and subsequent phosphorylation of many myocyte proteins (troponin I, voltage L-type calcium channels, cardiac ryanodine receptor) involved in cardiac contractility (Rockman et al., 2002; Xiang and Kobilka, 2003). The importance of β_1_-ARs in cardiovascular regulation has been shown in β_1_-AR knockout mice. Many did not survive past embryo stage and if they did, an increased heart rate in response to isoprenaline was found to be absent while the inotropic response to exercise/agonist stimulation was still present (Rohrer et al., 1996). On the other hand, transgenic mice with overexpression of β_1_-ARs develop marked hypertrophy and increased contractility initially, but this is soon followed by the onset of heart failure (Engelhardt et al., 1999). β_2_-ARs have some similarities with β_1_-ARs in regulating contractility by utilizing G_s_-proteins and the AC pathway with the eventual downstream release of calcium from L-type Ca^2+^ channels (Salazar et al., 2007). They differ in that β_2_-ARs can additionally couple to G_i_ (Daaka et al., 1997). Mice with overexpression of the β_2_-AR at 60-fold exhibited enhanced basal cardiac function without increased mortality when followed for 1 year. However, after 100-fold or more overexpression they developed a fibrotic cardiomyopathy and heart failure which increased in severity with overexpression level (Liggett et al., 2000). Knockout mice, however, display a relatively normal phenotype but develop a higher degree of hypertension in response to stress (exercise, adrenaline) when compared to control mice (Chruscinski et al., 1999). This may indicate the importance of the inhibitory G-coupled protein pathway in prolonged adrenergic stimulation (Salazar et al., 2007). β_3_-ARs are expressed the least in the heart and their role in cardiovascular regulation is a little less certain with inotropic effects in response to agonists in mice overexpressing human β_3_-ARs; however, a negative inotropic response in the human heart has been seen (Gauthier et al., 1996; Kohout et al., 2001). β_3_-ARs are also up-regulated in human heart failure (Moniotte et al., 2001). Studies on hPSC-CMs have shown that β-AR responses are well developed in cardiomyocytes derived from hESCs and hiPSCs (Ali et al., 2004; Dambrot et al., 2014). Furthermore, the expression of both β_1_-AR and β_2_-AR has also been established, with β_1_-AR being suggested as the predominant subtype for the late stage of cardiac differentiation (Wu et al., 2013, 2015).

#### Angiotensin, muscarinic, and adenosine receptors

Other GPCRs also present on hPSC-CMs are ATs and muscarinic receptors as shown by expression studies and agonist responses in these cells. Muscarinic receptors reduce spontaneous beating rate in hPSC-CM from an early time after differentiation, although the muscarinic receptor subtype has not been delineated (Brito-Martins et al., 2008). Adenosine can produce similar effects through the A_1_-R in adult ventricular or atrial cardiomyocytes (Headrick et al., 2013) but to date no published data exists for hPSC-CMs. We have shown previously that angiotensin acting via G_q_ can produce only a small increase in cell size in hESC-CM despite a robust increase in the expression of both atrial natriuretic factor and B-type natriuretic peptide (ANF and BNP; Földes et al., 2008, 2014).

#### Endothelin receptors

Endothelin receptors, specifically the endothelin-A (ET_A_) receptor, are subtypes of receptors involved in cardiac remodeling/hypertrophy. ET_A_ is expressed in the cardiovascular system and has a plethora of roles including vasoconstriction, tachycardia, positive inotropy and hypertrophy (Concas et al., 1989; Salazar et al., 2007; Bupha-Intr et al., 2012). In addition, ET receptors are up-regulated in chronically failing human hearts (Asano et al., 2002; Salazar et al., 2007). In hPSC-CM, ET_A_ induces hypertrophic gene expression; such as BNP and ANF (Carlson et al., 2013; Földes et al., 2014). The exact downstream signaling mechanisms have not yet been published.

#### Disease modeling with hPSC-CM

Predominant manifestations of pathology investigated in hPSC-CM are acute, including depressed contraction, electrophysiological alterations and arrhythmia, or longer-term, such as aberrant morphology, hypertrophy and increased susceptibility to cell death. While the acute characteristics have strong superficial similarities to adult cardiomyocytes, a clear difference in long term viability is seen in the prolonged survival in culture of hPSC-CM (>1 year) compared to adult cells (~2 days). This of course is one of the main attractions of hPSC-CM as a model system. Although proliferation rates in hPSC-CM are initially far higher than in adult cardiomyocytes, these drop rapidly around 1 month after differentiation, as the sarcomere structure develops (Földes et al., 2011). Morphology is initially less organized, but can develop with time or physical cues. The nature of the differences between adult and hPSC-CM phenotypes and the consequent limitations for modeling are discussed further below.

One pathological process where GPCR signaling plays a prominent role is cardiac hypertrophy. This is an adaptive response and is characterized by a thickening of cardiac myocytes. Physiological hypertrophy occurring in pregnancy and athletes is not detrimental and results in normal or enhanced heart function. In contrast pathophysiological hypertrophy, which can be caused by pressure overload in response to hypertension, myocardial infarction or other inherited conditions, leads to cardiac dysfunction and increased mortality. Approaches have been taken to model hypertrophy *in vitro* using hPSC-CM.

The predominant α-ARs in the myocardium are α_1_-ARs (Brückner et al., 1985) and stimulation with catecholamines induces pathological cardiac hypertrophy (Rokosh et al., 1996; Zhong and Minneman, 1999). We have previously reported an increase in cell size in hESC-CM in response to the α-AR agonist PE (Földes et al., 2011), attributed to activation of p38 MAPK signaling pathways. As described above, α_1A_-AR gene expression was lost upon differentiation in hESC-CM (and hiPSC-CM), while α_1B_-AR was up-regulated and mediated the hypertrophic response (Földes et al., 2014). In addition ET_A_, Ang II and cyclic stretch also increased cell size in hESC-CM (Földes et al., 2011, 2014) with corresponding increases in ANF expression. β_2_-AR stimulation did not induce cellular hypertrophy.

Hypertrophic responses in hiPSC-CM remain controversial. In contrast to hESC-CM, we found hiPSC-CM to be unresponsive to PE, with cell size (assessed by high content automated microscopy) and ANF expression remaining unchanged (Földes et al., 2014). In addition, ET-1 and Ang II did not produce significant increases in cell size and correspondingly increased ANF and BNP expression was only seen in response to ET_A_. Hypertrophic modeling in commercially available hiPSC-CM assays have been described which rely on detection of ANF expression in response to ET_A_ (Aggarwal et al., 2014). In other studies, mild increases in hiPSC-CM size (~10% or less) have been seen with PE, and up to 25% with ET-1 (Carlson et al., 2013; Tanaka et al., 2014). In addition, enhanced myofibrillar disarray and nuclear factor of activated T-cells (NFAT) nuclear translocation were also reported (Zhi et al., 2012). There is conflicting data for the presence of cardiomyocyte hypertrophy in response to β-adrenergic stimulation. We found no increase in hiPSC-CM size (Földes et al., 2014), whereas Zhi and colleagues found the opposite (Zhi et al., 2012). It has been reported that serum containing media causes hypertrophy in hESC-CM and hiPSC-CM, which could explain the lack of cellular hypertrophy in response to hypertrophic stimuli in some studies (Dambrot et al., 2014). This was found not to be the explanation in our study (Földes et al., 2014); the difference between hESC-CM and hiPSC-CM was caused rather by an imbalance in anti-hypertrophic signaling. It was also found that a combination of inhibitors could restore the PE response in hiPSC-CM.

A number of hiPSC-CM disease models have a hypertrophic phenotype, including LEOPARD syndrome and hypertrophic cardiomyopathy (HCM). Patient-derived HCM hiPSC-CM exhibit increased basal cell size compared to controls (Lan et al., 2013; Tanaka et al., 2014). β-AR stimulation exacerbates cellular hypertrophy in HCM cells (Lan et al., 2013). A hiPSC-CM model of LEOPARD of syndrome exhibits a hypertrophic phenotype, displaying increased cell size and nuclear located NFAT (Carvajal-Vergara et al., 2010). It still remains to be determined whether HCM patient-derived cardiomyocytes or control cells treated with hypertrophic stimuli are the best model to use for the study of hypertrophy. Furthermore, a greater understanding of hypertrophic signaling in hPSC-CM is required to ensure conclusions drawn from these models are physiologically relevant.

In addition to hypertrophy, disease models have also provided further insight into pathological mechanisms involving GPCRs (Table 2). Patient-derived dilated cardiomyopathy (DCM) hiPSC-CMs display an increased susceptibility to stress. Desensitization of the β-AR response was observed in DCM hiPSC-CM both basally and in response to acute noradrenaline treatment (Sun et al., 2012). This goes against the current understanding of β-AR desensitization as an acquired characteristic of prolonged sympathetic stimulation in the heart failure patient. Either the troponin mutation has some mechanistic link to the control of β-AR function, or there is a co-inherited β-AR variant in this group of patients: either option is intriguing. In addition, long term β-AR stimulation resulted in sarcomeric disorganization and decreased inotropic and chronotropic responses. In patient-derived HCM hiPSC-CM, β-adrenergic stimulation also exacerbated the observed abnormal calcium handling and arrhythmia (Lan et al., 2013). In long QT syndrome (LQTS2 and LQTS1) hiPSC-CM models, arrhythmia was observed in response to β-adrenergic stimulation, which could be prevented using β-AR blockers (Tseng et al., 2006; Matsa et al., 2011). This correlates with clinical observations, where β-AR blockers are routinely used to treat such conditions. The majority of these disease models originate from patient derived-hiPSC, but the trisomy 21 model described by Bosman and co-workers utilizes patient hESC-derived cardiomyocytes (Bosman et al., 2015). In this study trisomic cells showed an increased β-AR response to isoprenaline in comparison to euploid control (Bosman et al., 2015).

**Table 2:** **PSC-CM models of cardiac-related diseases**.

**Condition**	**Cell source**	**Mutation**	**GPCRs investigated**	**Phenotype**	**References**
LEOPARD syndrome	hiPSC	Protein tyrosine phosphatase, non-receptor type 11 gene (PTPN11)	Unknown	Entigines, electrocardiographic abnormalities, ocular hypertelorism, pulmonary valve stenosis, abnormal genitalia, retardation of growth and deafness	Carvajal-Vergara et al., 2010
Long QT syndromes (LQTS)	hiPSC	A614V missense mutation in the KCNH2 gene, c.A2987T (N996I) KCNH2 mutation, KCNH2 G1681A mutation	β-AR	Delayed repolarization of the heart, arrhythmia	Itzhaki et al., 2011; Matsa et al., 2011; Bellin et al., 2013
Catecholaminergic polymorphic ventricular tachycardia (CPVT)	hiPSC	p.F2483I mutation in ryanodine receptor 2	β-AR	Ventricular arrhythmia	Kujala et al., 2012; Novak et al., 2012; Zhang et al., 2013
Dilated cardiomyopathy (DCM)	hiPSC	Point mutation R173W in exon 12 of troponin T2 gene	β-AR	Non-ischemic cardiomyopathy	Sun et al., 2012; Karakikes et al., 2015; Wu et al., 2015
Hypertrophic cardiomyopathy (HCM)	hiPSC	Missense mutation on exon 18 of the β-myosin heavy chain gene (Arg663His)	β-AR	Non-ischemic cardiomyopathy, enlargement of the cardiac cells	Lan et al., 2013; Han et al., 2014
Arrhythmogenic right ventricular cardiomyopathy (ARVD)	hiPSC	c.2484C>T mutation in PKP2	β-AR	Ventricular arrhythmia	Kim et al., 2013
Timothy syndrome	hiPSC	Missense mutation in the L-type calcium channel CaV1.2	Unknown	Heart QT prolongation, arrhythmias, structural cardiac defects, webbing of fingers and toes and autism spectrum disorders	Yazawa et al., 2011; Song et al., 2015
Barth syndrome	hiPSC	Mutation of gene encoding tafazzin	Unknown	Cardiomyopathy, neutropenia, underdeveloped skeletal musculature and muscle weakness, growth delay, cardiolipin abnormalities	Wang et al., 2014
Diabetic cardiomyopathy	hiPSC	N/A	Endothelin, β-AR	Cardiomyopathy	Drawnel et al., 2014
Duchenne muscular dystrophy (DMD)	hiPSC	Mutation in DMD gene encoding dystrophin	Unknown	Muscle degeneration and premature death	Lin et al., 2015
Down's syndrome	hESC	Trisomy 21	β-AR	Delayed physical growth, facial features, and intellectual disability	Bosman et al., 2015

### Vascular derivatives

ECs form a single-cell monolayer lining the blood vessels. Their essential functions include the ability to regulate vascular tone, vascular permeability, angiogenesis, platelet function and inflammatory responses (Michiels, 2003). ECs are involved in inflammation and interact closely with leukocytes. GPCRs expressed in these cells play a key role in sensing the presence of chemoattractants, transducing signals that lead to the production of cytokines and regulating vascular permeability (Table 3). ECs are therefore critical for vascular homeostasis, and cellular dysfunction is strongly associated with an increased risk of cardiovascular events (Lerman and Zeiher, 2005). Generating novel ECs is a powerful *in vitro* technique to study cellular responses under various culture conditions and to develop constructs for tissue engineering. PSC-ECs are suggested to have many of the properties of endogenous ECs and their phenotypes are being investigated to determine whether characteristics of vascular disease can be reproduced *in vitro*.

**Table 3:** **GPCRs present in human endothelial cells**.

**GPCR**	**Ligand**	**Role**	**References**
Platelet activating factor receptor (PAF)	Platelet activating factor (PAF)	Vascular permeability, increasing gap formation between endothelial cells	Handley et al., 1984
Histamine receptor (H)	Histamine	Vascular permeability	Bakker et al., 2002
Protease activated receptor (PAR)	Thrombin	Vascular permeability, cellular differentiation, migration, and proliferation of VSMC, angiogenesis and vascular development	Patterson et al., 2001
S1PR	S1P	Stabilization of the endothelial barrier	English et al., 1999
CXCR4	SDF	Chemotaxis	Hoggatt et al., 2013
AT	Ang	Vasodilation, growth inhibition, vascular tone	Pueyo and Michel, 1997
ET	ET-1	Vasoconstriction, vascular homeostasis	Kedzierski and Yanagisawa, 2001

VSMCs have a plethora of roles in the cardiovascular system from producing extracellular matrix proteins which provide elasticity and the ability to withstand high circulating pressures to being involved in arterial repair and regulation of vascular tone. They are primarily contained in the media layer of blood vessels (Lacolley et al., 2012). The sympathetic nervous system regulates vascular tone and primarily acts on VSMCs via ARs. β_2_-ARs agonists cause vasodilation and hypotension while α-AR (α_1∕2_) agonists cause vasoconstriction (Barbato, 2009). Like PSC-ECs, PSC-VSMCs are an interesting area of research and are being used in tissue engineering strategies as well as an avenue for studying human diseases of the vascular smooth muscle (Xiang and Kobilka, 2003; Salazar et al., 2007). Little has been reported regarding GPCR function in PSC-VSMC apart from a contractile response to carbachol, and even then the muscarinic subtype was not identified (Dash et al., 2015).

#### CXCR4 receptor

The SDF-CXCR4 axis plays an important role in stem cell trafficking, chemotaxis, engraftment, and therapeutic angiogenesis (Hoggatt et al., 2013). CXCR4 is required for normal vascularization of the small intestines and mesentery branching (Tachibana et al., 1998). Murine iPSC-ECs express abundant CXCR4 protein intracellularly, but not on the cell surface. When iPSC-ECs were systemically delivered, these did not home to the site of hindlimb ischemia *in vivo*. It was also noted that iPSC-ECs did not respond to chemotactic gradients of SDF. Overall this suggests that these cells retain an immature phenotype (Huang et al., 2013). Because GPCR proteins are typically expressed at low levels in endogenous tissues, the use of proteomic profiling approaches for identifying further endothelial-specific GPCRs proves problematic.

#### Angiotensin receptor

Ang II stimulates VSMC contraction and aldosterone release with consequent sodium retention. It also stimulates the production of ECM proteins and is pro-inflammatory (Wollert and Drexler, 1999). Ang II is the main mediator of this pathway and signals primarily through the AT_1_ receptor (Touyz and Schiffrin, 2000). AT_1_ receptors are upregulated in response to hypertensive rats and hypertrophic stimuli (Suzuki et al., 1993). In contrast, they are down-regulated in systolic heart failure (Rogg et al., 1996). Overexpressing AT_1A_ in mice resulted in hypertrophy and fibrosis of myocardial tissue (collagen deposition; Paradis et al., 2000). AT_1A_ deficient mice, however, were more resistant to the effects of myocardial ischemia with less ventricular dilatation and fibrosis and a better recovery in LV function 4 weeks after infarction (Harada et al., 1999). To date, the effect of Angiotensin receptors and stem cells has only been investigated in mice. AT_1_R stimulation has been found to enhance not only the proliferation but also the differentiation of undifferentiated pluripotent stem cells into mesodermal progenitor cells (Ishizuka et al., 2012).

#### Endothelin receptor

The endothelin pathway also has a regulatory role in hPSC-ECs. Differentiation of hESC into endothelial cells (hESC-ECs) can be a potential source of cells and endothelial factors for ischemic diseases by supporting angiogenesis and vasculogenesis (Burdon et al., 1999; Lesman et al., 2010). Protocols for new ECs from hPSC generated cells with high initial clonal proliferative potential with self-repopulating activity and *in vivo* vessel-forming ability have been devised (Ingram et al., 2004; Földes et al., 2010; James et al., 2010). However, a number of differences between hPSC-derived cells and adult ECs have been noted. For example, we found that hESC-EC failed to release the GPCR ligand endothelin-1 (ET-1) at levels comparable to human aortic ECs or to blood outgrowth ECs (Reed et al., 2014). However, in a separate study it was demonstrated that hiPSC-EC were able to upregulate ET-1 expressionin response to atheroprone flow (Adams et al., 2013).

#### Disease modeling with PSC-ECs and PSC-VSMC

In contrast to myocytes, limited studies using disease models in EC and VSMC are available. They have been used for vascular repair: the first model using ESC-ECs has recently been evaluated in myocardial infarction and hindlimb ischemia as a therapeutic option to promote angiogenesis and neovascularization (Cho et al., 2007; Yu et al., 2009). iPSC-ECs derived from diet-induced obese mice exhibits endothelial dysfunction and may not be suitable for therapeutic transplantation in a hindlimb ischemia model. Furthermore, the administration of statins reversed endothelial dysfunction both *in vitro* and *in vivo* (Gu et al., 2015).

Recently, hiPSC lines were differentiated from patients with supravalvular aortic stenosis (William's syndrome). The VSMCs displayed a blunted maturation profile with fewer organized smooth muscle α-actin filament bundles networks and also had a higher proliferation rate (a hallmark of the disease). Reversion to a wild type phenotype was achieved by the addition of recombinant elastin protein or enhancing small GTPase RhoA signaling (Ge et al., 2012). In addition, hiPSC-VSMCs have been derived from patients with Hutchinson-Gilford progeria syndrome (HGP), a disease carrying a lamin A mutation and increased progerin levels, leading to premature aging and early mortality by myocardial infarction/stroke. The differentiated VSMCs contained high levels of progerin and also exhibited a new phenotype, calponin-1 staining inclusion bodies in the cytoplasm. Additionally, the VSMCs had nuclear abnormalities and increased DNA damage compared to controls (Zhang et al., 2011). To date, the GPCR related signaling of these human diseases have not been clarified.

## Summary: focussed targeting of GPCR signaling in human cardiovascular system

hPSCs show potential as a platform for both studying disease as well as an autologous source of cells for possible transplantation therapy (Lee et al., 2010a). Particularly for cardiomyocytes, where adult cells are difficult to manipulate in culture and options for cell lines are severely limited, the advent of disease-specific hiPSC-CM represents a great step forward. Differentiation methods are improving in efficiency and reproducibility. However, models should never be accepted uncritically, and a more sophisticated dissection of their fidelity has begun to appear. One major limitation is the greater resemblance of hPSC-CM to immature cardiomyocytes, although this may also be a reflection of the general differences induced by 2D cell culture. This could present a problem for models of late onset diseases. A wide array of approaches are being undertaken to improve maturation of these cells in an attempt to provide better models of disease. These include; prolonged time in culture (Ivashchenko et al., 2013), application of triiodothyronine (T3; Lee et al., 2010b; Ivashchenko et al., 2013; Yang et al., 2014; Ribeiro et al., 2015), manipulation of culture substrate (Rao et al., 2013; Tallawi et al., 2015), 3 D culture (Schaaf et al., 2011) and long term electrical pacing (Lieu et al., 2013; Hirt et al., 2014). hPSC-EC also display an immature phenotype (Huang et al., 2013), which still requires further investigation.

Another limitation is the use of correct controls, particularly for disease models. Obtaining control material from familial relatives of patients can prove difficult, which makes drawing solid conclusions from these disease models problematic. Gene editing technology approaches, such as the clustered regularly interspaced short palindromic repeats (CRISPR) system and zinc finger nucleases(ZFN) have arisen as useful tools to generate control lines on the same genetic background as the diseased cells. Alternatively, recreation of disease causing mutations in wild type cell lines using these technologies is an option and will allow multiple mutations to be compared in a more controlled manner. Additionally, identifying pharmacologically-relevant phenotypes in these models is important. It also remains to be determined whether monogenic disease and pharmacological models are comparable in cardiovascular diseases. The expected phenotype is not always present, for example in cardiac hypertrophy (Földes et al., 2014), which may be a limitation of the *in vitro* models. Modeling conditions with a broader tissue based-phenotype including scar formation, fibrosis and tissue disarray are also not possible using hPSC-CM in 2D culture, although the reconstruction of 3D tissue models may allow advances in this area. In particular, a greater understanding of GPCR signaling in hPSC-CM is needed to ensure accurate disease modeling and to determine suitability for use in pharmaceutical compound screening. More focussed investigation into the expression profile and functional characterization of GPCRs in PSC-derived cardiovascular cells is required to establish their resemblance to *in vivo* models.

## Author contributions

ND, NH, RJ, GF contributed to the conception of the review and wrote the manuscript. SH critical revised and final approved the review.

## Funding

This paper was supported by the British Heart Foundation RM/13/1/30157 and Fondation Leducq Shapeheart Network 5 CVD 03.

### Conflict of interest statement

The authors declare that the research was conducted in the absence of any commercial or financial relationships that could be construed as a potential conflict of interest.

## Abbreviations

7TM receptors: seven-transmembrane domain receptors
α-ARs: α-adrenergic receptor
AC: adenylyl cyclase
ACE: angiotensin converting enzyme
ANF: atrial natriuretic factor
APC: adenomatosis polyposis coli
AT_1_: angiotensin receptor1
β-AR: β-adrenergic receptor
BBB: blood-brain barrier
BMP4: bone morphogenetic protein 4
BNP: B-type natriuretic peptide
cAMP: 3′, 5′-Cyclic adenosine monophosphate
CK1a: casein kinase 1a
CRISPR: clustered regularly interspaced short palindromic repeat
DCM: dilated cardiomyopathy
DMD: Duchenne muscular dystrophy
DVL: Disheveled
EC: endothelial cell
ECM: extracellular matrix
ERK1/2: extracellular signal-regulated kinases 1/2
ET_A_: endothelinreceptor A
FGF: fibroblast growth factor
FZD: Frizzled receptor
GDP: guanosine diphosphate
Gi: guanine-coupled inhibitory protein
GSK3: glycogen synthase kinase 3
GPCR: G-protein coupled receptors
Gs: guanine-coupled stimulatory protein
GTP: guanosine triphosphate
HCM: hypertrophic cardiomyopathy
hESC: human embryonic stem cells
HF: heart failure
HGP: Hutchinson-Gilford progeria syndrome
hPSC: human pluripotent stem cells
JNK: c-Jun amino (N)-terminal kinase
IP3: inositol-1, 4, 5-triphosphate
LEF: lymphoid enhancer factor
LPA: lysophosphatidic acid
LQT: long QT syndrome
mESC: murine ESC
MI: myocardial Infarction
NFAT: nuclear factor of activated T-cells
PAF: platelet activating factor
PDGF: platelet derived growth factor
PE: phenylephrine
PI3-K: phosphoinositide 3-kinase
PKA: protein kinase A
PKC: protein kinase C
PLC: phospholipase C
PP2A: protein phosphatase 2A
PSC-EC: pluripotent stem cell-derived endothelial cells
PTX: pertussis toxin
S1P: sphingosine 1 phosphate
SDF1: stromal cell derived factor 1
SFRP: secreted frizzled related protein
SM: smooth muscle
T3: triiodothyronine
TCF: T-cell factor
TGF-β: transforming growth factor β
VEGF: vascular endothelial growth factor
VSMC: vascular smooth muscle cell
ZFN: zinc finger nuclease.
